# Effects of PREPARE, a Multi-component, School-Based HIV and Intimate Partner Violence (IPV) Prevention Programme on Adolescent Sexual Risk Behaviour and IPV: Cluster Randomised Controlled Trial

**DOI:** 10.1007/s10461-016-1410-1

**Published:** 2016-05-03

**Authors:** Catherine Mathews, Sander M. Eggers, Loraine Townsend, Leif E. Aarø, Petrus J. de Vries, Amanda J. Mason-Jones, Petra De Koker, Tracy McClinton Appollis, Yolisa Mtshizana, Joy Koech, Annegreet Wubs, Hein De Vries

**Affiliations:** 1 Health Systems Research Unit, South African Medical Research Council, P.O. Box 19070, Tygerberg, Cape Town, 7505 South Africa; 2 Adolescent Health Research Unit, Division of Child & Adolescent Psychiatry, University of Cape Town, Cape Town, South Africa; 3 Department of Health Promotion, Caphri Research Institute, Maastricht University, Maastricht, The Netherlands; 4 Department of Health Promotion and Development, University of Bergen, Bergen, Norway; 5 Division of Mental Health, Norwegian Institute of Public Health, Oslo, Norway; 6 Division of Child & Adolescent Psychiatry, University of Cape Town, Cape Town, South Africa; 7 Department of Health Sciences, University of York, York, England, UK

**Keywords:** Adolescents, HIV prevention, Intimate partner violence, Sexual risk behaviour, Randomised controlled trial

## Abstract

Young South Africans, especially women, are at high risk of HIV. We evaluated the effects of PREPARE, a multi-component, school-based HIV prevention intervention to delay sexual debut, increase condom use and decrease intimate partner violence (IPV) among young adolescents. We conducted a cluster RCT among Grade eights in 42 high schools. The intervention comprised education sessions, a school health service and a school sexual violence prevention programme. Participants completed questionnaires at baseline, 6 and 12 months. Regression was undertaken to provide ORs or coefficients adjusted for clustering. Of 6244 sampled adolescents, 55.3 % participated. At 12 months there were no differences between intervention and control arms in sexual risk behaviours. Participants in the intervention arm were less likely to report IPV victimisation (35.1 vs. 40.9 %; OR 0.77, 95 % CI 0.61–0.99; t(40) = 2.14) suggesting the intervention shaped intimate partnerships into safer ones, potentially lowering the risk for HIV.

## Introduction

Globally, HIV is ranked second among the leading causes of death among adolescents [1]. Among adolescents and youth in South Africa, there has been little progress in preventing new infections. Although declining somewhat, the HIV prevalence and incidence among young South Africans 15–24 years remains high, especially among women [2–4]. In the Western Cape, South Africa, the setting of this study, HIV is still the leading cause of premature mortality (http://www.mrc.ac.za/bod/WC2010Report.pdf), and adolescents commonly report an early sexual debut and unprotected sex [5]. These behaviours increase the risk of sexually transmitted infections (STI) including HIV.

In South Africa, adolescents’ intimate relationships are marked by a high incidence of violence [6]. Sexual violence and intimate partner violence (IPV) increase the risk of STIs including HIV among women [7]. This implies that to be effective, HIV prevention interventions should include a focus on preventing sexual violence and IPV. In the Western Cape and Limpopo provinces of South Africa, cluster RCTs of school-based HIV prevention interventions without a focus on IPV, failed to demonstrate an impact on the timing of sexual debut or condom use among the younger adolescent participants (average age 13 years) and also failed to impact on IPV [5]. However, in the Eastern Cape, a community-based HIV and IPV prevention programme for adolescents 16 years and older, which included a substantial focus on IPV, showed a beneficial impact on STI incidence and male IPV perpetration [8].

In our cluster randomised controlled trial, PREPARE, conducted among young adolescents (average age 13 years) in the Western Cape, we evaluated an HIV prevention programme that included a focus on IPV and sexual violence reduction. In the current trial we tested the hypothesis that the PREPARE programme would 1. delay sexual debut; 2. increase the use of condoms; 3. decrease the number of sexual partners among young adults. A secondary objective was to assess the effect of the intervention on IPV, and (not reported here) the three-year incidence of conceptions among female participants.

## Methods

### Intervention

The intervention was multi-component, comprising an educational programme, a school health service and a school safety programme, and described in detail in Table 1. The educational programme consisted of 21 sessions delivered once a week, immediately when school ended, in the school premises. The session duration ranged from 1 to 1.5 h. Sessions included up to 25 participants, and the education methods were interactive and skills-based. The programme was built upon the Respect4U programme, an IPV and HIV prevention intervention which was developed based on the Jewkes conceptual framework [9], and piloted-tested among Grade 8 students in nine typical Western Cape high schools over 3 years [10]. It was also informed by social cognition models including the Reasoned Action Framework [11] and the I-Change theoretical model [12]. Staff employed by the PREPARE project, who had been screened for positive gender norms and comfort with sexuality education and condom demonstrations, facilitated groups of up to 25 participants. The facilitators received a two-week training course and subsequent weekly training, supervision and session preparation support.

**Table 1:** The PREPARE intervention objectives and sample activities

Educational programme		
Topic and number of sessions	Objectives	Sample activities
Values and aspirations related in intimate relationships (1)	Meet the facilitator and learn about the programme Identify personal values and aspirations including how they want to treat people and be treated	Students complete a worksheet to design a “roadmap” to their chosen life goals Group discussion about relationships and their place in the roadmap
Assertive communication (2)	Identify four styles of communication and their consequences Develop assertive communication skills for sexual decision making	Students practice assertive communication to convey a wish to a sexual partner
Gender power inequities (2)	To critically analyse the dominant social ideas about gender power and roles To explore the kind of man or woman they want to be.	Group discussion of experiences of gender norms and gender inequality in home life/intimate relationships
Relationships (6)	To identify the characteristics of a caring relationship To identify qualities they value in an intimate partner To identify, and develop skills to respond to relationship problems To develop skills to end relationships respectfully and safely	Read custom-made photo-novella and discuss in a group the relationship problems experienced by the adolescent characters: alcohol, poor communication, pressure to have sex, IPV Enact resolutions to the problems through role-plays
Sexual decision-making (4)	To develop motivations and skills to delay sex, use condoms and reduce sex partners: Learn about positive and negative consequences of having sex Develop action plans to prevent having sex when they are not ready Identify risk behaviours for HIV, STI and unwanted pregnancy Critically analyse the risks of multiple partnerships, intergenerational partnerships, and transactional sex Develop skills to use a condom	Complete a worksheet to develop a set of personal criteria for assessing their own readiness to have sex Play a game to learn the steps in using a condom
IPV and sexual violence (4)	Recognize types of relationship violence and warning signs Understand the reasons people use violence and leading to intimate partner violence control to manipulate others Reflect on their own values and aspirations in relation to violence Understand the laws related to violence and sexual violence, and the legal support services Demonstrate risk monitoring and safety planning skills	Read a story in which a girl is forced to have sex by her boyfriend, and through discussion identify the underlying factors, the triggers and the opportunity factors leading to IPV
Support for victims of IPV and sexual violence (1)	Develop empathy towards victims of violence and learn how to support them Understand the importance of seeking help if a victim of violence, and to learn how to seek help	Read a story in which a girl is forced to have sex by her boyfriend and discuss issues of power, blame, responsibility and human rights violations
Creating lasting change Consolidating lessons learned (1)	To consolidate lessons learned To reflect on their ability to act as agents of change within their schools and communities	Complete and discuss a worksheet focusing on “What am I going to do to be more respected and respectful?”

School health service		
Activity and participants	Objectives	Sample activities
“Health check” offered to each PREPARE participant	To increase adolescent access to sexual and reproductive health (SRH) education, services and social support	SRH education, screening and referral for SRH problems Screening and referral for psychosocial problems Follow-up consultation

School safety programme (1)		
Activity and participants	Objectives	Sample activities
School safety training School teams from each intervention school, comprising principal, teachers, school safety officer, parent representatives, local police officer	To reduce acceptability and prevalence of IPV and sexual violence in the school To raise awareness of the relevant laws concerning sexual violence To develop skills to implement a participatory school safety audit and safety plan	Presentation of the laws regarding sexual violence Presentation of concepts of participatory safety audit and plan Small group-work to plan school safety audit

School safety programme (2)		
Activity and participants	Objectives	Sample activities
Photovoice (five 2-h sessions) 20 randomly selected PREPARE participant volunteers	To empower students to be the driving force in improving physical, emotional and sexual safety at school To influence school safety policy and prompt changes to address violence in schools	Risk mapping of unsafe situations and places in school Take photographs to portray safe and unsafe situations and places Present to forum of principals, teachers, parents, police officers, and community stakeholders

The school health service (SHS) was implemented in collaboration with the Western Cape Department of Health, the City of Cape Town Health Department, and the Desmond Tutu HIV Foundation. A nurse from the public clinic nearest to the school delivered the service in the school premises, once a week immediately after school ended. The service was modeled on the new South African Integrated School Health Policy (http://www.health-e.org.za/2013/10/24/integrated-school-health-policy/), was free, and involved SRH education, identification of need for SRH services or commodities and referral for such services or commodities to the nearest community clinic, where they were provided free of charge. Some clinics were also able to send a health promoter to assist with health education. There are no known randomized controlled trials of the impact of SHS on adolescent SRH, however they are considered to be an accessible and acceptable strategy [13].

The school safety programme comprised two initiatives. School safety teams were invited to a two-day training at a central venue, conducted by the PREPARE team with the Centre for Justice and Crime Prevention (CJCP) (a non-government organisation). We implemented “Photovoice”, a carefully piloted programme [14], for twenty, randomly selected students at each school, facilitated by two PREPARE researchers.

### Control Condition

Participants in the control schools received school as usual, which excluded the after-school programme, the school health service and the safety programme.

### Sampling

The study population comprised adolescents in Grade 8 (average age 13) in public high schools in the Western Cape. The sample size was calculated to have the power to show an intervention effect on the annual incidence of the primary outcome of sexual debut. We estimated 83 % of the participants would report they had not yet had their sexual debut at baseline [5]; 17 % of participants in the control arm would have their sexual debut during the PREPARE follow-up period of 1 year [5]; an intra-class correlation coefficient (ICC) of 0.06; and 20 % participant drop-out after 1 year. We used the Hayes and Bennett formula [15] to estimate that we would need 19 schools in each arm, with 62 of 75 participants pre-sexual debut at baseline in each school, to show a 50 % relative reduction in 1 year incidence of sexual debut (17 % in control schools and 8.5 % in intervention schools) with 80 % power for a 2-sided test with a significance level of 5 %.

We sampled 40 schools to allow for one school per arm to drop out of the study. They were randomly sampled using the database of 359 public high schools in the Western Cape Province. Of the 359 schools, before sampling we excluded: 1 school with Grade 12 pass rates below 40 % (indicating their inability to deliver on their core educational mandate); 33 schools with pass rates above 97 % (indicating well-resourced schools already able to offer students the types of interventions proposed by PREPARE); 67 schools in two of the eight districts situated more than 3-h drive from Cape Town; and schools participating in other HIV prevention trials. After sampling 40 schools, we found that some of them did not have the required 75 Grade 8 students. We paired four small schools to create two sampling units, and we randomly sampled two more schools from the database, generating a total sample of 42 schools (40 sampling units).

### Randomisation and Masking

We stratified schools into two strata based on the Grade 12 pass rate which we assumed was an indication of how well the school functioned and its potential ability to benefit from the PREPARE programme. We found that pass rate was correlated with the amount of school fees charged, indicating it is also a reflection of socioeconomic status. Before allocating schools to conditions, we invited each of the 42 schools to participate, and all accepted.

To ensure allocation sequence concealment, a statistician at the South African Medical Research Council who did not have any knowledge of the schools, allocated them within each stratum to intervention and control arms of the study. Using a spreadsheet, he ordered the school names randomly within each stratum and then used a random number generator to give each school a number. Within each stratum, he allocated the schools with the lowest random numbers to the intervention arm, and the other half to the control arm. We selected classes randomly so that we would obtain a sample of at least 75 assenting students per school with signed parental consent.

**Fig. 1:**
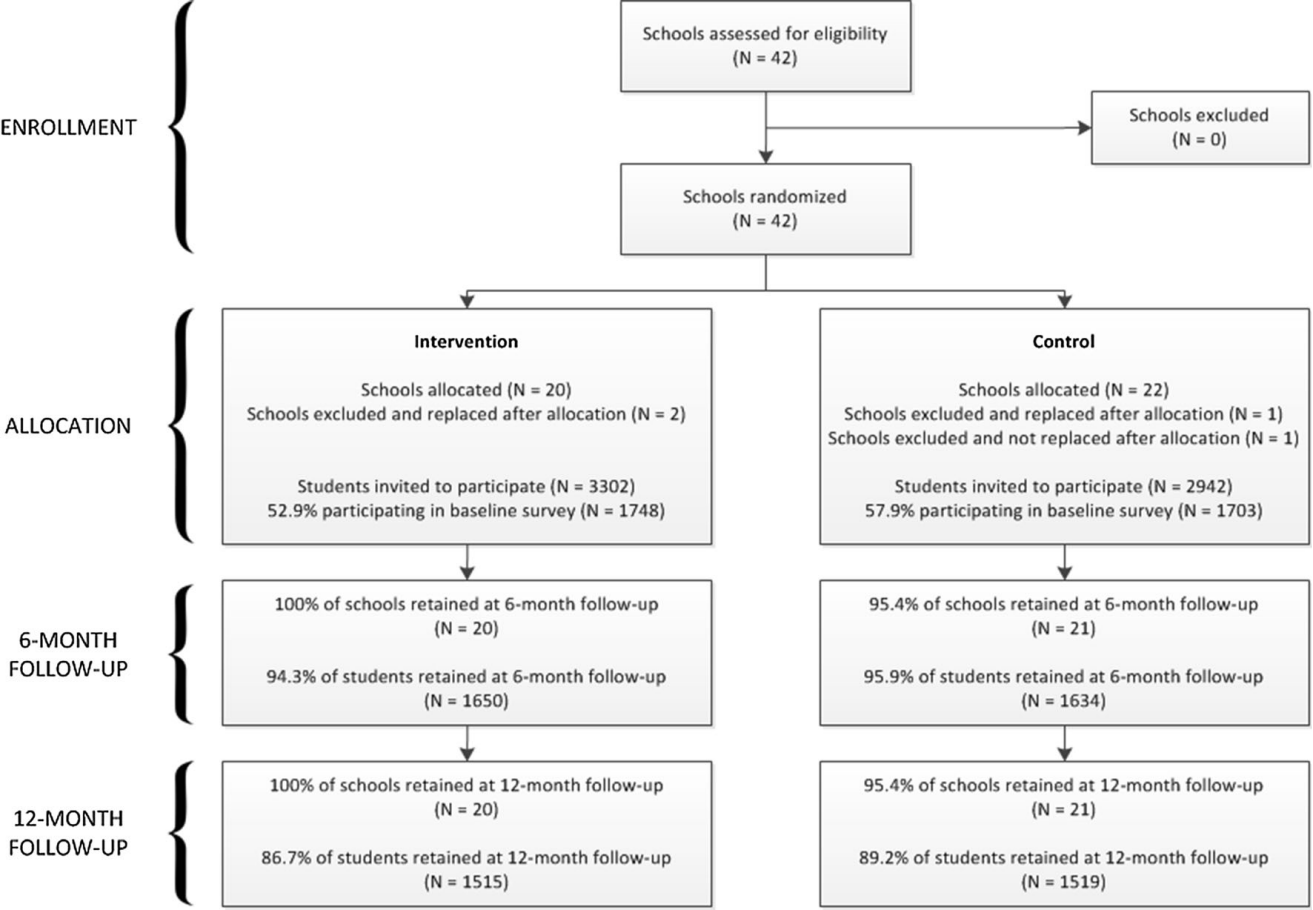
Screening and follow-up of study participants from screening and baseline enrolment through follow-up 1 (6 months) and follow-up 2 (12 months)

After allocation assignment, the director of one of the Department of Health sub-districts requested that we exclude three of the participating schools because they had been identified for a Department of Health intervention. One had been allocated to the comparison arm and two to the intervention arm. We replaced these schools with randomly selected schools from the database, and the statistician randomly assigned one to the control arm and two to the intervention arm. When we were making plans to conduct the baseline survey, one school in the control arm withdrew from the study because they were unable to find time for the survey. We did not replace this school (Fig. 1). We were not able to mask intervention assignment.

### Recruitment of Participants

We sampled all Grade 8 classes in the selected schools, or, in large schools, we randomly selected Grade 8 classes to ensure at least 75 participants. We invited 6244 Grade 8 students in the 41 schools to participate in the PREPARE trial and 3451 (55.3 %) returned signed parental consent forms, gave assent and participated in the baseline survey in February and March 2013 (Fig. 1). In the intervention schools, participants were given an invitation to the after-school programme after they had completed the baseline survey. To encourage attendance, we offered refreshments at each session and small stationary gifts at selected sessions. Each participant had a “loyalty card” which was stamped at each session or nurse consultation. We gave R50.00 (~ US$5) supermarket gift voucher and a certificate for those who attended at least 15 sessions.

### Measures

The instrument comprised a paper, self-administered questionnaire for each of the surveys (baseline and 6 and 12 months post-baseline) with a set of common questions across time points. Each question was provided in the three languages commonly spoken, and printed in full colour in an adolescent-friendly format resembling a “teen magazine”. The questionnaire was informed by an instrument used in a previous study [5] and formative qualitative research we conducted with adolescents in six schools to identify the salient attitudes, beliefs, and social norms that served as barriers and facilitators to safe sexual behaviour. It was piloted (including conducting group cognitive interviews after students completed it in a classroom setting) and revised during 2011 to ensure maximum face validity and reliability of measures. The surveys were conducted during school hours and completion of the questionnaire took on average 45 min. At the 6- and 12-month surveys, we visited the school up to three times in an attempt to increase retention of absent participants. In the 12-month survey, we surveyed students who had left the participating school at their new school, at their home or at a convenient venue in the community in which they lived. (We had obtained consent to make such arrangements and had collected contact details of all participants). The behavioural outcome variables and the variables measuring theorized motivational determinants and their psychometric properties are described in Table 2. The items to measure IPV were adapted from the WHO multi-country study [16].

**Table 2:** PREPARE behavioural outcome variables, theorized motivational variables and their psychometric properties

Variable	Number of items	Items	Scoring	Alpha
Sexual behavior				
Sexual debut		Have you ever had vaginal sex? Have you ever had anal sex?	h	NA
Sexual debut including oral sex		Have you ever had vaginal sex? Have you ever had anal sex? Have you ever had oral sex?	h	NA
Unwilling first sex (defined by b, c, or d)		The first time I had sex, was it Something I wanted Something I did not want Something I was forced to do against my will I was raped		NA
Regretted first sex (defined by a or d)		Think back to the first time you had sex and tell us what you feel about it now I wish I’d waited longer before having sex I wish I’d not waited so long It was about the right time It shouldn’t have happened at all Don’t know		NA
Vaginal sex frequency		How many times in the past 6 months have you had vaginal sex?	i	NA
Anal sex frequency		How many times in the past 6 months have you had anal sex?	i	NA
Gave money or gifts for sex		In the past 6 months, have you given someone gifts or money in exchange for sex?	h	NA
Received money or gifts for sex		In the past 6 months, have you had sex because you expected to get money, food, drinks or other gifts?	h	NA
Number of sex partners		How many people have you had sex with in your life?		NA
Condom use at last sex (defined by a, b, or c) Contraception use at last sex (defined by b, c, d, or e)		The last time you had sex, what did you or your partner do to prevent pregnancy? Condom only Condom with pill Condom with injection Injection alone Pill alone	h	NA
Condom use frequency		In the past 6 months, how often did you use a condom when having sex?	j	NA
Carrying condoms		Have you carried a condom with you in the past 6 months?	h	
Intimate partner violence (IPV)				
IPV victimization (defined by at least once on a, b, c or d)		In the past 6 months how often has a boyfriend or girlfriend Insulted you or humiliated you or made you feel bad about yourself? Threatened to hurt you? Hit, pushed, kicked, choked or burned you? Forced you to have sex with him/her?	k	NA
IPV perpetration (defined by at least once on a, b, c or d)		In the past 6 months, how often have you Insulted or humiliated a girlfriend or boyfriend or made them feel bad? Threatened to hurt a boyfriend or girlfriend? Hit, pushed, kicked, choked or burned a boyfriend or girlfriend? Forced a boyfriend or girlfriend to have sex with you?	k	NA
Theorized motivational variables for sexual behaviour				
Knowledge condom use	3	Does a condom have an expiry date? Should a man leave a bit of air at the top of the condom when putting it on? Is it true that the only time a person should use a condom is when they have sex with someone for the first time?	a	NA
Knowledge HIV/AIDS	5	If you have sex only once with a person who is HIV positive, can you become infected with HIV? If you kiss with a person who is HIV positive, can you become infected with HIV? If you have anal sex with a person who is HIV positive, can you become infected with HIV? Is it true that a person who is strong and health can be HIV positive? When a girl uses contraceptive pills or the injection for family planning, does this protect her against STIs?	a	NA
Risk susceptibility	2	If I do not use a condom when having sex, my risk of getting a STI will be.. If I do not use a condom when having sex, my risk of HIV infection will be..	b	0.73
Risk severity	2	If I got a STI, I would find this… If I was infected with HIV, I would find this..	c	0.82
Attitude: pros condom use	4	If I use a condom when I have sex this will Show that I take responsibility for myself Make me less worried about having sex Show that I respect my partner Show that I am sexually experienced	d	0.63
Attitude: pros delaying sex	4	Waiting until I am older before I have sex will: Help me achieve my life’s goals Help prevent me from getting hurt emotionally Please my parents Lower my risk of getting HIV	d	0.73
Attitude: cons condom use	**4**	If I use a condom when I have sex this will Be unacceptable for me because of my religion Feel unnatural t me Takes too much effort Will make me feel nervous/awkward	d	0.78
Attitude: cons delaying sex	**4**	Waiting until I am older before I have sex will: Make me look uncool Will be frustrating for me Make my partner frustrated with me Make me look unsuccessful	d	0.82
Social norm condom use	**4**	Most of my friends think that I should use a condom when I have sex My parents/caregivers think that I should use a condom when I have sex Most of my other family members think that I should use a condom when I have sex x My boyfriend or girlfriend thinks that I should use a condom when I have sex	d	0.86
Social norm delaying sex	**4**	Most of my friends think that I should wait until I am older before I have sex My parents/caregivers think that I should wait until I’m older to have sex Most of my other family members think that I should wait until I’m older before I have sex My boyfriend or girlfriend thinks that I should wait until I am older before I have sex	d	0.76
Self-efficacy condom use^e^	5	Using a condom when I have a steady partner is… Using a condom when I feel sexually excited is… Using a condom when I am drunk is… Using a condom when I do not feel comfortable (when I am shy) is… Going to a clinic to get condoms is…	e	0.73
Self-efficacy delaying sex^e^	4	If I have been drinking alcohol, waiting until I’m older before I have sex is… If my partner is older than me, waiting until I am older before I have sex is… If someone offers me money or gifts, waiting until I am older before I have sex with that person is… If I am deeply in love, waiting until I am older before I have sex is…	e	0.79
Action planning condom use^d^	4	I plan to use a condom when I have a steady partner I plan to always keep a condom in a safe place at home I plan to discuss condom use with each partner before we have sex I plan to get a new condom when I have only one left	d	NA
Action planning delaying sex^d^	4	I plan to not have sex when I have had more than one glass of alcohol I plan to not get a partner who is much older than me I plan to not have sex for money, food or gifts I plan to find other ways of showing my love (instead of having sex	d	NA
Intentions delaying sex	1	I intend to have sex during next month	d	NA
Intentions delaying sex	1	I intend to have sex during the next 6 months	d	NA
Intentions condom use	1	I intend to use a condom the next time I have sex	d	NA

^a^Percentage of correct answers

^b^Scale from ‘very low’ (1) to ‘very high’ (5)

^c^Scale from ‘not serious’ (1) to ‘very serious’ (5)

^d^Scale from ‘strongly disagree’ (1) to ‘strongly agree’ (5)

^e^Scale from ‘very difficult’ (1) to ‘very easy’ (5)

^f^Scale from ‘never’ (0), ‘once’ (1) to ‘more than once’ (2)

^g^Scale from ‘never’ (1), ‘sometimes’ (2) to ‘often’ (3)

^h^‘No’ (0) and ‘yes’(1)

^i^Scale from ‘not at all’ (1), ‘1 time’ (2), ‘2-5 times’ (3), ‘6-10 times’ (4), ’11-20 times’ (5) to ‘more than 20 times’ (6)

^j^Scale from ‘never’ (1) to ‘every time’ (5)

^k^Never (0), Once or more than once (1)

We measured participants’ attendance at each of the 21 weekly after-school education sessions over 6 months between February and September 2013, using a register of participants, and roll calls at each session. School nurses kept registers of the participants who consulted them and individual patient records. Each participant’s attendance records were linked with the data from their baseline survey.

### Process Evaluation

Process data were collected in the 6- and 12-month follow-up questionnaires. To assess fidelity to the intervention, facilitators of the education sessions were observed at random, unannounced times by two independent observers, for a total of two sessions each in different schools. Their performance was scored from 1 to 5 on 13 aspects of session facilitation. A score of 1 indicated that aspect had not been achieved, and a score of 5 indicated that aspect had been excellent. The 13 aspects were grouped into following three dimensions: interaction with learners (for example, listened attentively); session fidelity (for example, implemented all activities); facilitation skills (for example, encouraged learners’ involvement). Each of the 14 facilitator’s scores was summed and averaged for the 4 observations.

### Analysis

We computed the dimensionality and internal consistency of the scales measuring the motivational constructs and then calculated mean scores per construct. We performed a baseline descriptive analysis of the participants’ demographics, self-reported sexual risk behaviour and scores on the motivational constructs in intervention and comparison arms. At the 6 and 12 months follow-ups, we report the estimates in the intervention and comparison groups for the three primary sexual behaviour outcomes and five other sexual behaviour outcomes; the two secondary IPV outcomes and four other variables measuring sexual coercion; and 17 motivational constructs related to the primary outcomes. Regression was undertaken to provide outcomes at 6 and 12 months with odds ratios for dichotomous variables and coefficients for continuous variables, adjusted for baseline demographics (age, gender, socio-economic status), the baseline measure in question, and clustering using the complex samples approach in SPSS Version 20. These analyses were carried out among the full sample, the subsample that attended at least 1 session, and the subsample that attended at least 10 sessions (50 %) in order to be able to detect the effect of attendance rate on outcomes. In addition to these complete case analyses, all analyses were repeated on a data file that had missing values on latent factors imputed by applying the expectation–maximisation algorithm (EM). The EM algorithm uses maximum likelihood estimators and is considered one of the most favoured ways to impute missing data [17].

## Results

### Response Rate and Retention

We sampled 6244 adolescents in 41 schools, and 3451 (55.3 %) obtained signed parental consent and assented to participate (Fig. 1). The non-responders included 69 students and 281 parents who declined, and the remaining were students who did not bring back signed parental consent forms. Retention at 6 months was 94.1 % and at 12 months 87.9%.

### Exposure at the School Level

We implemented the after-school educational programme in all 20 intervention schools, but in two schools we were unable to complete it. In one of the two, the programme was interrupted by a religious fast. In the other, the school could no longer find a free afternoon for the sessions. We trained nurses and health promoters for 17 of the 20 intervention schools, and the school health service operated in 17 schools. The public health services did not have capacity to provide school health nurses for two of the remaining schools and in one, the school was not able to allocate an afternoon session for the school health service. The school safety teams of 18 of the 20 intervention schools participated in the school safety training, and we trained 53 school safety team delegates in total. None of the schools implemented participatory safety audits or develop safety plans during the 6 months following the safety training. We implemented the Photovoice programme in 10 of the 20 intervention schools. In two of these schools we did not complete the implementation of Photovoice because students did not feel safe taking photographs in the school premises (one school) and because attendance had dwindled (one school). An overview of exposure at the school level shows that seven schools were exposed to all four components of the intervention (educational sessions, school health service, school safety training and Photovoice); seven schools had all components except Photovoice; two schools had all components except the health service; two schools had all components except the safety training; and two schools were exposed to only the educational sessions and the school safety training.

### Exposure at the Individual Participant Level

In intervention schools the mean (M) attendance of PREPARE education sessions was 8.02 sessions (Standard Deviation (DS): 7.44; range 0–21) and was higher among girls (M: 8.8; SD 7.5) than boys (M: 6.9; SD 7.2). We recorded 363 (40.8 %) females and 330 (51.2 %) males attended fewer than 5 sessions; 272 females (30.6 %) and 141 males (21.9 %) attended more than 15 sessions. The PREPARE school nurse was visited by 17.3 % of the trial participants in intervention schools, (14.9 % of boys and 18.7 % of girls). Attendance of the 20 randomly selected participants in Photovoice varied between 7 and 20 students.

### Fidelity to Programme

The average facilitator performance scores ranged from 32.3 to 58.7 out of a maximum score of 65. Eleven of the fifteen facilitators scored above 50/65 indicating that the sessions were conducted with a moderately high degree of fidelity by the majority of facilitators. Three facilitators scored below 50. On closer inspection 2 of these 3 facilitators scored low on interaction with learners, yet had acceptable scores on session fidelity and facilitation skills. The other facilitator had low scores on all 3 dimensions, but none of the scores was below 25.

### Acceptability to Students

The intervention was highly acceptable to the participants in the intervention arm with three-quarters (1003; 75.1 %) rating the PREPARE after-school sessions as “excellent” or “very good”; 262 (19.5 %) rating “good” or “fair”; and 32 (2.3 %) rating “bad” to “extremely bad”. The remaining 39 (2.9 %) selected “I did not attend”.

### Effect Evaluation

At baseline there were no significant differences between participants in the intervention and comparison arms of the study (Table 3). The adjusted outcomes at first follow-up (Table 4) showed no significant differences by study arm in sexual debut (10.5 vs. 9.3 %; AOR: 1.09; 95 % CI 0.82–1.45), self-reported condom use at last sex (79.2 vs. 83.0 %; AOR: 0.69; 95 % CI 0.34–1.37), number of sexual partners in the past 6 months (0.56 vs. 0.31; B: 0.35; 95 % CI −0.37–1.07). Participants in the intervention arm had significantly better condom and HIV/AIDS knowledge scores compared with the control arm. The intracluster correlations for the primary and secondary outcomes were: sexual debut: 0.016; condom use at last sex: insufficient statistical power to calculate; number of sexual partners: 0.049; IPV victimization: 0.022; IPV perpetration: 0.024.

**Table 3:** Sample characteristics at baseline

	Control		Intervention		Difference	*p*
	Mean	SD	Mean	SD		
Age	13.7	1.07	13.71	0.99	F(1,40) < 1	Ns
Gender (% male, N)	37.9 %	(628)	41.5 %	(706)	F_a_(1,40) = 1.86	Ns
SES	5.99	1.65	5.98	1.68	F(1,40) < 1	Ns
Risk susceptibility	3.59	1.19	3.64	1.19	F(1,40) < 1	Ns
Risk severity	3.99	1.17	4.05	1.13	F(1,40) < 1	Ns
Knowledge condom use	0.28	0.24	0.29	0.24	F(1,40) < 1	Ns
Pros condom use	3.83	0.76	3.85	0.74	F(1,40) < 1	Ns
Cons condom use	2.78	0.90	2.74	0.94	F(1,40) < 1	Ns
Social norm condom use	4.26	0.84	4.28	0.79	F(1,40) < 1	Ns
Self-efficacy condom use	2.95	0.89	2.96	0.89	F(1,40) < 1	Ns
Action planning condom use	4.13	0.91	4.15	0.90	F(1,40) < 1	Ns
Knowledge HIV/AIDS	0.56	0.28	0.58	0.26	F(1,40) < 1	Ns
Pros delaying sex	3.99	0.83	4.03	0.88	F(1,40) < 1	Ns
Cons delaying sex	2.57	1.02	2.51	1.07	F(1,40) < 1	Ns
Social norm delaying sex	4.34	0.78	4.30	0.79	F(1,40) < 1	Ns
Self-efficacy delaying sex	3.08	1.07	3.11	1.09	F(1,40) < 1	Ns
Action planning delaying sex	3.65	1.18	3.64	1.21	F(1,40) < 1	Ns
I intend to have sex during next month	1.86	1.27	1.92	1.31	F(1,40) < 1	Ns
I intend to have sex during the next 6 months	1.78	1.19	1.78	1.16	F(1,40) < 1	Ns
I intend to use a condom the next time I have sex	3.85	1.44	3.94	1.43	F(1,40) = 1.01	Ns
Primary outcomes						
Ever had sex (vaginal or anal)	22.0 %	(270)	23.3 %	(299)	F_a_(1,40) < 1	Ns
Ever had sex (vag., anal or oral)	29.5 %	(339)	31.0 %	(374)	F_a_(1,40) < 1	Ns
Vaginal sex frequency^a^	1.55	1.04	1.69	1.20	F(1,40) = 2.32	Ns
Anal sex frequency^a^	1.43	0.96	1.47	1.04	F(1,40) < 1	Ns
Lifetime number of sexual partners^a^	3.35	3.04	3.36	2.76	F(1,40) < 1	Ns
Condom use last intercourse^a^	87.7 %	(350)	88.2 %	(373)	F_a_(1,40) < 1	Ns
Condom use frequency^a^	1.87	1.45	1.80	1.41	F(1,40) < 1	Ns
Ever carried a condom	16.5 %	(261)	16.6 %	(273)	F_a_(1,40) < 1	Ns
Contraception use (excl cu)^a^	25.6 %	(69)	27.1 %	(81)	F_a_(1,40) < 1	Ns
Secondary outcomes						
IPV victim (%, N)	45.5 %	(626)	46.1 %	(632)	F_a_(1,40) < 1	Ns
IPV perpetrator (%, N)	26.4 %	(352)	27.4 %	(363)	F_a_(1,40) < 1	Ns
Unwilling at first sex^a^	31.4 %	(58)	25.7 %	(53)	F_a_(1,40) = 2.04	Ns
Gave money or gifts for sex^a^	8.1 %	(25)	12.3 %	(42)	F_a_(1,40) = 2.05	Ns
Received money or gifts for sex^a^	9.1 %	(29)	10.3 %	(36)	F_a_(1,40) < 1	Ns

^a^Only students who indicated to have had oral, anal or vaginal sex were included

* *p* < 0.05; ** *p* < 0.01

**Table 4:** Intervention effects 6 months after baseline

	Prevalence/mean at 6 months		Unadjusted effect estimate			Adjusted effect estimate		
	Control (M, SD)	Intervention (M, SD)	B/OR	95 % CI	Test statistic	B/OR	95 % CI	Test statistic
Risk susceptibility	3.73 (1.10)	3.87 (1.15)	0.13	−0.06 to 0.33	F(1,40) = 1.88	0.12	−0.03 to 0.27	F(1,40) = 2.53
Risk severity	4.11 (1.05)	4.14 (1.13)	0.02	−0.20 to 0.24	F(1,40) < 1	−0.01	−0.15 to 0.13	F(1,40) < 1
Knowledge condom use	0.29 (0.25)	0.37 (0.27)	0.07***	0.04 to 0.11	F(1,40) = 18.10	0.07***	0.04 to 0.10	F(1,40) = 27.27
Pros condom use	3.87 (0.73)	3.92 (0.75)	0.04	−0.05 to 0.12	F(1,40) < 1	0.03	−0.05 to 0.10	F(1,40) < 1
Cons condom use	2.62 (0.88)	2.56 (0.97)	−0.07	−0.20 to 0.07	F(1,40) < 1	−0.03	−0.12 to 0.06	F(1,40) < 1
Social norm condom use	4.36 (0.75)	4.35 (0.81)	−0.02	−0.10 to 0.07	F(1,40) < 1	−0.02	−0.08 to 0.05	F(1,40) < 1
Self − efficacy condom use	3.29 (1.23)	3.39 (1.33)	−0.01	−0.14 to 0.12	F(1,40) < 1	−0.02	−0.13 to 0.09	F(1,40) < 1
Action planning condom use	4.18 (0.84)	4.23 (0.83)	0.06	−0.02 to 0.14	F(1,40) = 1.80	0.04	−0.03 to 0.11	F(1,40) = 1.51
Knowledge HIV/AIDS	0.57 (0.28)	0.62 (0.27)	0.06**	0.02 to 0.10	F(1,40) = 8.38	0.05**	0.02 to 0.08	F(1,40) = 10.84
Pros delaying sex	4.06 (0.81)	4.14 (0.82)	0.06	−0.06 to 0.18	F(1,40) = 1.13	0.07	−0.02 to 0.16	F(1,40) = 2.75
Cons delaying sex	2.38 (0.98)	2.28 (1.03)	−0.09	−0.25 to 0.07	F(1,40) = 1.42	−0.07	−0.16 to 0.02	F(1,40) = 2.49
Social norm delaying sex	4.36 (0.73)	4.30 (0.76)	−0.05	−0.16 to 0.06	F(1,40) < 1	−0.03	−0.10 to 0.04	F(1,40) < 1
Self − efficacy delaying sex	3.25 (1.04)	3.23 (1.07)	−0.03	−0.17 to 0.11	F(1,40) < 1	−0.03	−0.11 to 0.05	F(1,40) < 1
Action planning delaying sex	3.79 (1.15)	3.83 (1.14)	0.05	−0.07 to 0.16	F(1,40) < 1	0.05	−0.04 to 0.15	F(1,40) = 1.22
I intend to have sex during next month	1.86 (1.24)	1.88 (1.27)	0.02	−0.22 to 0.26	F(1,40) < 1	−0.01	−0.13 to 0.12	F(1,40) < 1
I intend to have sex during the next 6 months	1.79 (1.16)	1.80 (1.17)	0.02	−0.18 to 0.22	F(1,40) < 1	0.01	−0.11 to 0.12	F(1,40) < 1
I intend to use a condom the next time I have sex	4.07 (1.32)	4.11 (1.32)	0.06	−0.11 to 0.24	F(1,40) < 1	0.02	−0.13 to 0.16	F(1,40) < 1
Primary outcomes								
Sexual debut (%, N, OR)	9.3 % (143)	10.5 % (165)	1.14	0.84 to 1.55	t(40) < 1	1.09	0.82 to 1.45	t(40) < 1
Sexual debut (incl oral) (%, N, OR)	11.0 % (173)	13.2 % (211)	1.23	0.95 to 1.59	t(40) = 1.61	1.19	0.95 to 1.49	t(40) = 1.55
Vaginal sex frequency^a^	1.75 (1.09)	1.85 (1.27)	0.04	−0.13 to 0.20	F(1,40) < 1	0.12	−0.03 to 0.26	F(1,40) = 2.67
Anal sex frequency^a^	1.40 (1.00)	1.42 (0.93)	0.03	−0.10 to 0.15	F(1,40) < 1	0.02	−0.09 to 0.14	F(1,40) < 1
Number of sexual partners, past 6 months^a^	0.31 (2.63)	0.56 (2.42)	0.65	−0.08 to 1.23	F(1,40) < 1	0.35	−0.37 to 1.07	F(1,40) < 1
Condom use last intercourse (%, N, OR)^a^	83.0 % (88)	79.2 % (95)	0.80	0.50 to 1.30	t(40) < 1	0.69	0.35 to 1.37	t(40) = 1.20
Condom use frequency^a^	2.34 (1.61)	2.32 (1.63)	−0.04	−0.40 to 0.32	t(40) < 1	0.09	−0.24 to 0.42	F(1,40) < 1
Ever carried a condom (%, N, OR)	16.4 % (234)	17.4 % (254)	1.09	0.82 to 1.45	t(40) < 1	1.04	0.81 to 1.34	t(40) < 1
Contraception use (excl condoms) (%, N, OR)^a^	18.8 % (26)	23.8 % (36)	0.97	0.61 to 1.56	t(40) < 1	1.22	0.65 to 2.28	t(40) < 1
Secondary outcomes								
IPV victim (%, N, OR)	38.7 % (423)	37.2 % (401)	0.94	0.72 to 1.23	t(40) < 1	0.90	0.71 to 1.14	t(40) < 1
IPV perpetrator (%, N, OR)	27.1 % (282)	27.5 % (281)	1.02	0.77 to 1.35	t(40) < 1	0.98	0.76 to 1.27	t(40) < 1
Unwilling at first sex (%, N, OR)^b^	32.2 % (48)	28.9 % (44)	0.86	0.54 to 1.36	t(40) < 1	0.85	0.71 to 1.02	t(40) = 1.05
Regretted first sex (%, N, OR)^b^	–	–	–	–	–	–	–	–
Gave money or gifts for sex (%, N, OR)^a^	8.6 % (13)	11.4 % (19)	1.61	0.81 to 3.22	t(40) = 1.39	1.24	0.55 to 2.78	t(40) < 1
Received money or gifts for sex (%, N, OR)^a^	6.8 % (11)	11.8 % (20)	2.28	0.97 to 5.36	t(40) = 1.95	2.15	0.87 to 5.30	t(40) = 1.72

Adjusted effects were adjusted for clustering, age, gender, SES, stratification and the baseline measure; * *p* < 0.05; ** *p* < 0.01; *** *p* < 0.001

^a^Only students who indicated to have had oral, anal or vaginal sex were included

^b^Only students who had their sexual debut during the study period were included

The adjusted outcomes at the 12-month follow-up (Table 5) showed no differences between intervention and control arms in 1-year incidence of self-reported sexual debut (12.7 vs. 12.0 %; AOR: 1.07; 95 % CI 0.83–1.40), self-reported condom use at last sex (80.6 vs. 83.5 %; AOR: 0.64, 95 % CI 0.33–1.25), number of sexual partners in the past 12 months (0.62 vs. 0.40; B: −0.03; 95 % CI −0.71 to 0.64). Participants in the intervention arm reported significantly better condom knowledge scores and lower rates of IPV in comparison to the control arm. Baseline rates of past 6-month IPV victimization were high, but these dropped in absolute terms, by over 10 % in the intervention arm and 4.6 % in the control arm. Participants in the intervention arm were more likely to report using contraception (other than condoms) than those in the control arm (28.1 vs. 22.1 %) but the difference was not statistically significant.

**Table 5:** Intervention effects 12 months after baseline

	Prevalence/mean at 12 months		Unadjusted effect estimate			Adjusted effect estimate			
	Control (M, SD)	Intervention (M, SD)	B/OR	95 % CI	Test statistic	B/OR	95 % CI	Test statistic	
Risk susceptibility	3.79 (1.10)	3.78 (1.20)	−0.01	−0.20 to 0.19	F(1,40) < 1	−0.01	−0.17 to 0.14	F(1,40) < 1	
Risk severity	4.12 (1.07)	4.08 (1.17)	−0.05	−0.28 to 0.18	F(1,40) < 1	−0.07	−0.21 to 0.08	F(1,40) < 1	
Knowledge condom use	0.30 (0.25)	0.39 (0.28)	0.09**	0.04 to 0.13	F(1,40) = 13.20	0.09***	0.05 to 0.13	F(1,40) = 19.00	
Pros condom use	3.89 (0.77)	3.91 (0.79)	0.01	−0.09 to 0.10	F(1,40) < 1	−0.01	−0.09 to 0.08	F(1,40) < 1	
Cons condom use	2.55 (0.90)	2.55 (1.00)	0.01	−0.12 to 0.14	F(1,40) < 1	0.01	−0.10 to 0.10	F(1,40) < 1	
Social norm condom use	4.35 (0.82)	4.34 (0.83)	−0.03	−0.12 to 0.06	F(1,40) < 1	−0.02	−0.09 to 0.06	F(1,40) < 1	
Self − efficacy condom use	3.23 (0.90)	3.25 (0.91)	0.02	−0.10 to 0.13	F(1,40) < 1	0.01	−0.09 to 0.11	F(1,40) < 1	
Action planning condom use	4.27 (0.86)	4.21 (0.89)	−0.06	−0.16 to 0.04	F(1,40) = 1.30	−0.06	−0.14 to 0.03	F(1,40) = 1.63	
Knowledge HIV/AIDS	0.56 (0.30)	0.60 (0.29)	0.04	−0.01 to 0.09	F(1,40) = 2.84	0.03	−0.01 to 0.07	F(1,40) = 3.19	
Pros delaying sex	4.15 (0.83)	4.18 (0.88)	0.03	−0.10– 0.17	F(1,40) < 1	0.02	−0.09 to 0.12	F(1,40) < 1	
Cons delaying sex	2.26 (1.08)	2.24 (1.12)	−0.02	−0.17 to 0.14	F(1,40) < 1	−0.01	−0.09 to 0.07	F(1,40) < 1	
Social norm delaying sex	4.36 (0.76)	4.31 (0.76)	−0.05	−0.14 to 0.04	F(1,40) = 1.09	−0.04	−0.11 to 0.02	F(1,40) = 1.78	
Self − efficacy delaying sex	3.45 (1.07)	3.38 (1.15)	−0.07	−0.21 to 0.07	F(1,40) = 1.07	−0.07	−0.16 to 0.02	F(1,40) = 2.44	
Action planning delaying sex	3.84 (1.17)	3.82 (1.19)	−0.01	−0.12 to 0.10	F(1,40) < 1	−0.01	−0.09 to 0.09	F(1,40) < 1	
I intend to have sex during next month	1.89 (1.27)	1.99 (1.34)	0.09	−0.20 to 0.37	F(1,40) < 1	0.07	−0.09 to 0.23	F(1,40) < 1	
I intend to have sex during the next 6 months	1.81 (1.15)	1.90 (1.25)	0.10	−0.14 to 0.34	F(1,40) < 1	0.09	−0.06 to 0.23	F(1,40) = 1.38	
I intend to use a condom the next time I have sex	4.04 (1.35)	4.07 (1.34)	0.02	−0.14 to 0.18	F(1,40) < 1	−0.02	−0.15 to 0.12		F(1,40) < 1
Primary outcomes									
Sexual debut (%, N, OR)	12.0 % (168)	12.7 % (177)	1.07	0.81 to 1.43	t(40) < 1	1.07	0.83 to 1.40		t(40) < 1
Sexual debut (incl oral) (%, N, OR)	13.4 % (192)	14.3 % (203)	1.08	0.81 to 1.44	t(40) < 1	1.09	0.84 to 1.41		t(40) < 1
Vaginal sex frequency^a^	1.91 (1.13)	2.00 (1.34)	0.08	−0.07 to 0.23	F(1,40) = 1.16	0.08	−0.09 to 0.25		F(1,40) < 1
Anal sex frequency^a^	1.40 (0.86)	1.51 (1.03)	0.12	−0.02 to 0.27	F(1,40) = 3.14	0.14	−0.02 to 0.26		F(1,40) = 2.65
Number of sexual partners, past 12 months ^a^	0.40 (2.82)	0.62 (2.75)	0.33	−0.06 to 0.71	F(1,40) = 2.88	−0.03	−0.71 to 0.64		F(1,40) < 1
Condom use last intercourse (%, N, OR)^a^	83.5 % (81)	80.6 % (83)	0.73	0.46 to 1.16	t(40) = 1.38	0.64	0.33 to 1.25		t(40) = 1.35
Condom use frequency^a^	2.67 (1.70)	2.56 (1.69)	−0.15	−0.47 to 0.17	F(1,40) < 1	−0.06	−0.42 to 0.31		F(1,40) < 1
Ever carried a condom (%, N, OR)	15.6 % (209)	15.6 % (210)	1.02	0.76 to 1.36	t(40) < 1	0.98	0.74 to 1.29		t(40) < 1
Contraception use (excl condoms) (%, N, OR)^a^	22.1 % (29)	28.1 % (34)	1.26	0.80 to 1.98	t(40) = 1.04	1.21	0.70 to 2.09		t(40) < 1
Secondary outcomes									
IPV victim (%, N, OR)	40.9 % (402)	35.1 % (342)	0.80	0.60 to 1.05	t(40) = 1.68	0.77*	0.61 to 0.99		t(40) = 2.14
IPV perpetrator (%, N, OR)	27.2 % (255)	27.6 % (254)	1.05	0.77 to 1.43	t(40) < 1	1.03	0.79 to 1.35		t(40) < 1
Unwilling at first sex (%, N, OR)^b^	26.4 % (48)	34.8 % (69)	1.49	0.83 to 2.68	t(40) = 1.39	1.57	0.88 to 2.80		t(40) = 1.58
Regretted first sex (%, N, OR)^b^	47.5 % (94)	44.9 % (101)	0.90	0.65 to 1.25	t(40) < 1	0.91	0.63 to 1.33		t(40) < 1
Gave money or gifts for sex (%, N, OR)^a^	10.4 % (14)	13.2 % (18)	1.88	0.79 to 4.51	t(40) = 1.46	1.96	0.83 to 4.61		t(40) < 1
Received money or gifts for sex (%, N, OR)^a^	9.2 % (13)	15.4 % (22)	1.64	0.59 to 4.51	t(40) = 1.59	1.80	0.66 to 4.93		t(40) = 1.12

Adjusted effects were adjusted for clustering, age, gender, SES, stratification and the baseline measure; * *p* < 0.05; ** *p* < 0.01; *** *p* < 0.001

^a^Only students who indicated to have had oral, anal or vaginal sex were included

^b^Only students who had their sexual debut during the study period were included

### Effect of Rate of Session-Attendance on Outcomes

Since attendance was voluntary and attendance rates ranged substantially among those in the intervention arm [18], additional subgroup analyses were performed firstly among participants who attended at least 1 intervention session, and secondly among participants who attended at least 10 intervention sessions (Table 6). After 6 months, participants who attended at least 1 session reported better knowledge of condoms and HIV and more positive attitudes towards delaying sex in comparison to the control arm. Participants, who attended more than 10 sessions, reported better knowledge of condoms and HIV, more positive attitudes towards condom use and delaying sex, less IPV victimization, but also more sexual debut in comparison to the control arm. After 12 months, participants who attended at least 1 session reported better knowledge of condoms and HIV, and less IPV victimisation. Participants who attended more than 10 sessions reported better knowledge of condoms and HIV, and more positive attitudes towards condoms and delaying sex (Table 6).

**Table 6:** Adjusted intervention effects 6 and 12 months after baseline stratified by attendance

	Results after 6 months						Results after 12 months					
	Attended at least 1 session			Attended >10 sessions			Attended at least 1 session			Attended >10 sessions		
	M (SD)	B/OR	95 % CI	M (SD)	B/OR	95 % CI	M (SD)	B/OR	95 % CI	M (SD)	B/OR	95 % CI
Risk susceptibility	3.90 (1.16)	0.12	−0.03 to 0.28	4.04 (1.12)	0.21^*^	0.05 to 0.38	3.85 (1.19)	0.02	−0.14 to 0.18	3.98 (1.17)	0.12	−0.07 to 0.30
Risk severity	4.17 (1.11)	−0.01	−0.14 to 0.15	4.31 (1.05)	0.07	−0.08 to 0.22	4.11 (1.17)	−0.05	−0.20 to 0.10	4.28 (1.08)	0.06	−0.09 to 0.21
Knowledge condom use	0.38 (0.27)	0.08^***^	0.05 to 0.11	0.43 (0.27)	0.13^***^	0.09 to 0.17	0.41 (0.28)	0.10^***^	0.06 to 0.14	0.46 (0.28)	0.15^***^	0.11 to 0.19
Pros condom use	3.93 (0.75)	0.04	−0.04 to 0.11	3.98 (0.74)	0.08^*^	0.01 to 0.16	3.93 (0.78)	0.02	−0.06 to 0.11	3.95 (0.80)	0.04	−0.05 to 0.13
Cons condom use	2.49 (0.98)	−0.08	−0.17 to 0.02	2.39 (0.97)	−0.14^**^	−0.24 to −0.04	2.51 (1.02)	−0.01	−0.12 −0.10	2.36 (0.98)	−0.14^**^	−0.24 to −0.05
Social norm condom use	4.38 (0.80)	0.01	−0.06 to 0.06	4.44 (0.77)	0.06	−0.09 to 0.13	4.38 (0.81)	0.02	−0.06 to 0.09	4.43 (0.76)	0.06	−0.02 to 0.14
Self − efficacy condom use	3.34 (1.32)	0.02	−0.09 to 0.13	3.43 (1.32)	0.10	−0.03 to 0.22	3.26 (0.92)	0.02	−0.09 to 0.12	3.25 (0.91)	0.01	−0.12 to 0.12
Action planning condom use	4.26 (0.82)	0.06	−0.01 to 0.12	4.35 (0.79)	0.14^***^	0.07 to 0.20	4.21 (0.90)	−0.06	−0.14 to 0.02	4.27 (0.88)	−0.01	−0.11 to 0.08
Knowledge HIV/AIDS	0.63 (0.27)	0.05^**^	0.02 to 0.08	0.67 (0.27)	0.09^***^	0.06 to 0.13	0.61 (0.29)	0.04^*^	0.01 to 0.08	0.64 (0.29)	0.07^**^	0.02 to 0.11
Pros delaying sex	4.18 (0.81)	0.10^*^	0.01 to 0.18	4.24 (0.78)	0.13^**^	0.04 to 0.23	4.20 (0.87)	0.02	−0.08 to 0.13	4.27 (0.84)	0.08	−0.03 to 0.18
Cons delaying sex	2.21 (1.02)	−0.11^*^	−0.20 to −0.02	2.08 (0.99)	−0.16^**^	−0.28 to −0.05	2.19 (1.12)	−0.04	−0.13 to 0.04	2.05 (1.09)	−0.11^*^	−0.19 − − 0.02
Social norm delaying sex	4.33 (0.75)	−0.02	−0.10 to 0.05	4.41 (0.70)	0.04	−0.04 to 0.12	4.33 (0.77)	−0.04	−0.11 to 0.03	4.38 (0.73)	0.01	−0.07 to 0.08
Self − efficacy delaying sex	3.27 (1.07)	−0.01	−0.09 to 0.08	3.35 (1.09)	0.03	−0.06 to 0.11	3.41 (1.16)	−0.06	−0.16 to 0.04	3.50 (1.17)	−0.01	−0.12 to 0.10
Action planning delaying sex	3.87 (1.14)	0.07	−0.03 to 0.18	3.92 (1.13)	0.10	−0.05 to 0.24	3.86 (1.16)	0.03	−0.07 to 0.13	3.91 (1.16)	0.05	−0.07 to 0.17
I intend to have sex during next month	1.85 (1.26)	−0.02	−0.14 to 0.11	1.69 (1.16)	−0.09	−0.21 to 0.03	1.95 (1.34)	0.05	−0.12 to 0.21	1.82 (1.28)	−0.02	−0.19 to 0.14
I intend to have sex during the next 6 months	1.79 (1.17)	0.01	−0.11 to 0.11	1.73 (1.71)	−0.02	−0.15 to 0.11	1.88 (1.24)	0.06	−0.08 to 0.21	1.73 (1.18)	−0.03	−0.17 to 0.11
I intend to use a condom the next time I have sex	4.13 (1.31)	0.03	−0.12 to 0.18	4.22 (1.26)	0.13	−0.03 to 0.29	4.12 (1.30)	0.05	−0.08 to 0.17	4.15 (1.31)	0.09	−0.07 to 0.24
Primary outcomes												
Sexual debut (%, N, OR)	11.0 % (139)	1.17	0.87 to 1.57	10.0 % (65)	1.11	0.80 to 1.54	12.5 % (142)	1.08	0.84 to 1.39	12.9 % (76)	1.15	0.86 to 1.53
Sexual debut (incl oral) (%, N, OR)	13.6 % (175)	1.26	0.99 to 1.61	13.9 % (91)	1.39^*^	1.05 to 1.85	14.1 % (162)	1.09	0.85 to 1.41	14.4 % (85)	1.16	0.87 to 1.55
Vaginal sex frequency^a^	1.84 (1.27)	0.10	−0.06 to 0.26	1.56 (1.07)	−0.11	−0.28 −0.06	1.94 (1.29)	0.01	−0.16 to 0.18	1.77 (1.10)	−0.11	−0.30 to 0.08
Anal sex frequency^a^	1.39 (0.93)	0.02	−0.11 to 0.14	1.27 (0.73)	−0.08	−0.19 to 0.04	1.45 (0.96)	0.09	−0.05 to 0.23	1.29 (0.77)	−0.04	−0.20 to 0.12
Number of sexual partners^a^	4.32 (3.36)	0.33	−0.35 to 1.02	3.53 (2.82)	−0.18	−0.92 to 0.56	4.15 (3.11)	−0.01	−0.77 to 0.75	3.05 (1.63)	−0.88	−2.17 to 0.41
Condom use last intercourse (%, N, OR)^a^	80.9 % (76)	0.71	0.35 to 1.44	83.3 % (20)	1.00	0.50 to 2.04	79.7 % (59)	0.65	0.33 to 1.27	73.9 % (17)	0.38	0.11 to 1.37
Condom use frequency^a^	2.35 (1.62)	0.15	−0.21 to 0.52	1.97 (1.49)	−0.10	−0.52 to 0.32	2.48 (1.67)	−0.08	−0.46 to 0.30	2.17 (1.57)	−0.34	−0.75 to 0.08
Ever carried a condom (%, N, OR)	17.2 % (204)	1.06	0.82 to 1.38	15.2 % (91)	0.98	0.71 to 1.34	14.6 % (159)	0.95	0.70 to 1.29	12.6 % (71)	0.83	0.57 to 1.21
Contraception use (excl condoms) (%, N, OR)^a^	23.9 % (27)	1.24	0.68 to 2.29	14.7 % (5)	0.76	0.31 to 1.90	23.3 % (21)	0.86	0.46 to 1.60	14.3 % (4)	0.61	0.24 to 1.54
Secondary outcomes												
IPV victim (%, N, OR)	34.7 % (297)	0.82	0.63 to 1.06	28.8 % (115)	0.66^*^	0.49 to 0.90	34.3 % (268)	0.76*	0.58 to 0.99	32.2 % (123)	0.73	0.53 to 1.01
IPV perpetrator (%, N, OR)	27.1 % (221)	0.99	0.77 to 1.27	22.8 % (87)	0.82	0.58 to 1.16	26.9 % (198)	1.01	0.77 to 1.31	25.6 % (93)	1.01	0.71 to 1.44
Unwilling at first sex (%, N, OR)^b^	27.2 % (34)	0.81	0.47 to 1.40	22.8 % (13)	0.72	0.34 to 1.51	35.7 % (56)	1.61	0.85 to 3.05	35.9 % (28)	1.72	0.65 to 4.54
Regretted first sex (%, N, OR)^b^	–	–	–	–	–	–	45.9 % (83)	0.94	0.63 to 1.42	45.8 % (44)	0.98	0.59 to 1.62
Gave money or gifts for sex (%, N, OR)^a^	8.8 % (11)	0.95	0.39 to 2.37	8.9 % (4)	1.96	0.90 to 4.26	8.5 % (9)	0.98	0.34 to 2.79	15.0 % (6)	2.11	0.82 to 5.39
Received money or gifts for sex (%, N, OR)^a^	7.9 % (10)	1.25	0.53 to 2.97	6.7 % (3)	2.20	0.86 to 5.64	15.2 % (17)	1.75	0.68 to 4.50	10.0 % (4)	1.14	0.37 to 3.54

Adjusted effects were adjusted for clustering, age, gender, SES, stratification and the baseline measure; * *p* < 0.05; ** *p* < 0.01; *** *p* < 0.001

^a^Only students who indicated to have had oral, anal or vaginal sex were included

^b^Only students who had their sexual debut during the study period were included

When all previously mentioned analyses were repeated using the expectation-maximisation algorithm to impute missing data, the results were similar: there were no additional significant effects or effects that ceased to exist in comparison to the complete case analyses (results not shown).

## Discussion

This evaluation of the PREPARE after-school behavioural HIV prevention programme, which included a focus on IPV prevention, provided no evidence that it reduced sexual risk behaviour. Despite beneficial effects on knowledge about HIV prevention, our evaluation gave no indication that participants in the intervention arm of the PREPARE trial were less likely to have their sexual debut, were more likely to use condoms or had fewer sexual partners than those in the comparison arm. There was also no indication that students who attended a greater number of education sessions reported less sexual risk behaviour than those in the control condition. Nor was there any suggestion that the “quality” of sexual debut was superior among the intervention participants: there were no differences between arms in an indicator of a “safe and good” sexual debut, defined by use of condoms and contraception at first sex, absence of coercion at first sex, and absence of regret about first sex (data not shown).

These findings are consistent with a previous trial of an in-school HIV prevention intervention in the Western Cape, the *SATZ* trial, which failed to impact sexual risk behaviour [5]. They are also consistent with the *SATZ* trial in Limpopo province [5]. However, they are not consistent with the findings of a meta-analysis of South African sexual risk reduction interventions among adolescents and youth, which found the interventions delayed sexual intercourse, increased condom use and reduced the number of sex partners [19]. The meta analysis suggested that features of interventions successful at delaying sexual intercourse and increasing condom use relative to the control condition were a focus on social norms, condom skills, gender inequalities and alcohol [19]. (All these factors were addressed in PREPARE).

One explanation for the failure of PREPARE in reducing sexual risk behavior is that, in the Western Cape setting, the contextual constraints on safe sexual behaviour might mitigate against any positive impact of the PREPARE intervention. Apart from IPV and sexual violence, there are numerous social and environmental factors which undermine adolescent sexual and reproductive health, which were not addressed in the PREPARE programme, and which might be particularly relevant in the study setting. For example, the PREPARE programme did not include interventions to ensure adolescents had safe, supportive homes, secure livelihoods, and social protection, factors important for adolescent sexual and reproductive health [1, 20]. However, these structural barriers to safe sexual behaviours apply to the other South African settings in which effective sexual risk reduction interventions have been demonstrated [19].

Another explanation for the absence of a beneficial effect on sexual behaviour is that adolescents might need to have a much greater intensity of exposure to the components of the PREPARE intervention, to achieve reductions in sexual risk behaviour. We have shown that exposure to the PREPARE after-school education sessions and school health service was sub-optimal. The findings of an HIV prevention trial in the Eastern Cape, South Africa suggest that greater intervention exposure, with a smaller facilitator-to-adolescent ratio are important factors in reducing sexual risk. Young adolescents who received intense exposure an in-school HIV/STI risk reduction intervention comprising twelve 1-h modules in small-groups, with very low participant-to-facilitator ratios (two facilitators to between nine and 16 participants), reported less unprotected sex over several subsequent years [21]. Further evidence in support of this explanation comes from a meta-analysis of the effects of South African sexual risk reduction interventions. It found that interventions were successful in delaying sexual intercourse and increasing condom use among youth when they used more facilitators to deliver the intervention and participants received a higher “dose” [19]. The most efficient way to ensure young adolescents have adequate exposure to HIV risk reduction interventions is to embed such interventions in the school curriculum. After-school programmes often do not achieve high levels of exposure because it is difficult to obtain high attendance rates and such programmes tend to selectively attract participants who are less vulnerable to adverse outcomes [18].

Our study suggests that behavioural HIV prevention programmes which include a focus on IPV prevention do indeed reduce self-reported intimate partner violence. In our evaluation, we found high baseline rates of IPV victimisation, a reduction in rates in both intervention and control arms over the course of the study but a significantly greater reduction in the intervention group. We observed an even greater impact on IPV victimisation among those with higher rates of education session attendance, compared with the control arm. The PREPARE trial is one of several adolescent IPV prevention programmes with evidence of beneficial effects on reported IPV [22, 23]. However, to our knowledge PREPARE is one of only two that have been trialled among adolescents in sub-Saharan Africa. The other trial conducted in this region of the world was Stepping Stones, conducted out of school, among older adolescents than those in the PREPARE trial [8]. The PREPARE study is one of several trials with a combined IPV/ HIV prevention focus [8, 24–26], but only PREPARE and Stepping Stones [8] were conducted predominantly among adolescents. PREPARE is the only one of these combination IPV/HIV trials that failed to impact both IPV and HIV risk (measured either by self-reported sexual risk behaviour or STI/HIV infection).

Given that the participants of the PREPARE trial had sub-optimal exposure to the PREPARE intervention components, how did the programme achieve a beneficial effect on IPV? We speculate that the explanation is that participants were more likely to be exposed to the PREPARE IPV intervention components than components focussing on sexual risk behaviour. The after-school education sessions were ordered so that the sessions addressing gender inequities and gender power came before the sessions addressing condom use, delaying sex and number of partners. Attendance was higher at the earlier sessions [18], and consequently more participants received exposure to the intervention content related to IPV, than content related to sexual risk behaviour. Furthermore, the school-level intervention (the school safety programme), focussed on reducing IPV and sexual violence and did not have any direct focus on sexual risk reduction.

## Limitations

Self-report measures are prone to bias and and biological measures provide the most convincing evidence of effects. The biological measure included in our trial (3 years incidence of conceptions among female participants) will only be available in 2016. Students who had signed parental consent and therefore participated in the study might not have been those most in need of interventions such as PREPARE. Therefore we might not have reached those who would benefit most from the programme. Our analyses of participants who attended more than one, or more than ten PREPARE education sessions compared with the control arm, are not based on an intention-to-treat analysis and are therefore potentially undermined by selection bias. We do not have the statistical power to perform gender-stratified analyses for the primary outcomes.

## Conclusions

Recent evidence shows adolescents are the only age group in which AIDS-related mortality is increasing, while in all other age groups it is decreasing [27]. Preventing new infections among adolescents and young people is a key goal in reaching the target of ending the AIDS epidemic by 2030. The PREPARE study aimed to prevent HIV among adolescents by reducing sexual risk behaviours and IPV. Despite adolescents’ low participation rate in the PREPARE intervention, we observed a reduction in IPV, which suggests the intervention shaped intimate partnerships into more safe and appropriate ones. Thus, we have demonstrated the potential of interventions such as PREPARE to have a beneficial effect on one of the factors which strongly affect adolescents’ risk of STIs and HIV. Reducing IPV is regarded as a critical goal for HIV prevention [28]. Women exposed to IPV have limited ability to assert their choices about sexuality [29] and are at a higher risk of incident HIV infection [28, 30]. Reducing IPV is thus likely to lower risk for HIV/STIs in the longer term.

The PREPARE intervention did not lead to any reductions in sexual risk behaviours related to HIV prevention, despite that the intervention focussed on, and had a beneficial effect on one of the key structural barriers to HIV prevention, gender violence. Young adolescents probably need more intense, sustained exposure to interventions such as PREPARE to have an impact on sexual risk behaviour. With after-school programmes, it is difficult to achieve such exposure. However, we believe our findings probably also imply that reducing HIV risk among adolescents requires interventions which address a greater range of structural, social and environmental barriers to behaviours that prevent HIV infection.
